# Feasibility, Preference, and Impact of a Rapid Multiplexed Point-of-Care Digital Innovation (AideSmart!) to Expedite Screening of Sexually Transmitted Blood-Borne Infections in At-Risk Populations in Canada: Cross-Sectional Study

**DOI:** 10.2196/55075

**Published:** 2024-10-18

**Authors:** Angela Karellis, Duncan Webster, Jean Boulanger, Kate Harland, Paige Feltmate, Stefanie Materniak, Gabriel Daunais-Laurin, Christine Mesa, Olivia Vaikla, John Kim, Nitika Pant Pai

**Affiliations:** 1 Infectious Diseases and Immunity in Global Health Research Institute of the McGill University Health Centre Montreal, QC Canada; 2 Centre for Research, Education & Clinical Care of At-Risk Populations Saint John, NB Canada; 3 REZO Montreal, QC Canada; 4 National Laboratory for HIV Reference Services Public Health Agency of Canada Winnipeg, MB Canada; 5 Faculty of Medicine McGill University Montreal, QC Canada

**Keywords:** sexually transmitted blood-borne infections, rapid multiplexed biomarker-based screening, digital strategy, feasibility, preference, impact

## Abstract

**Background:**

With the need to expedite the detection of multiple sexually transmitted blood-borne infections (STBBIs), there is an increased demand for digital innovations and tests that improve the efficiency of point-of-care testing in outreach community settings. Multiplexed testing is being offered to at-risk populations by frontline health care professionals.

**Objective:**

With this in mind, we evaluated AideSmart! (for health aides or health care workers), an integrated app and platform solution that enables multiplexed STBBI screening at the point of care, for feasibility, preference, accuracy, and impact. With AideSmart!, we provided trained health care workers with the ability to perform rapid multiplexed tests; offer STBBI pre- or posttest counseling; maintain quality assurance of testing; facilitate linkages to care; and enhance health care provider–patient communication, data documentation, and patient engagement through the multiplexed STBBI screening and linkage process. We evaluated the performance of multiplexed tests.

**Methods:**

In a cross-sectional study conducted during the COVID-19 pandemic, we recruited at-risk populations in Canada from community-based organizations in Montreal, Quebec, and Saint John, New Brunswick, with unknown serostatus for HIV, hepatitis C virus (HCV), and syphilis. Following orientation and pretest counseling with AideSmart!, we performed multiplexed tests, simultaneously screening for HIV, HCV, and syphilis, using 2 rapid investigational multiplexed tests (Chembio for HIV and syphilis and MedMira for HIV, HCV, and syphilis) followed by confirmatory testing from local and national laboratories.

**Results:**

Of the 401 participants, all (100%) accepted the AideSmart! multiplexed strategy: 59.4% (148/249) preferred multiplexed rapid tests over laboratory tests, and 56.6% (141/249) preferred receiving same-day test results. Rapid test results were obtained in 15 minutes (vs laboratory tests at 4-24 days). A total of 29 new infections (n=27, 93% HCV; n=1, 3% syphilis; and n=1, 3% HIV) were identified and treated within a week. Feasibility of the strategy (completion of testing and linkages to care) was at 76.1% (305/401). Health care professionals provided positive feedback and emphasized the importance of knowing one’s negative or positive serostatus, especially during a pandemic. Multiplexed rapid tests’ specificity (against laboratory reference standards) exceeded 98% (98.7%-100%) for all pathogens and devices. An electronic reader, used by the Chembio rapid test, enhanced sensitivity (HIV: 100%, 95% CI 79.4%-100%; syphilis: 86.8%, 95% CI 71.9%-95.6% [Chembio] vs HIV: 100%, 95% CI 78.2%-100%; HCV: 90.3%, 95% CI 80.1%-96.4%; and syphilis: 57.9%, 95% CI 40.8%-73.7% [MedMira]).

**Conclusions:**

The AideSmart! digital multiplexed rapid screening strategy for health care workers facilitated STBBI testing for multiple STBBIs and arranged for pre- or posttest counseling and rapid linkages with high feasibility and acceptability. Electronic readers enhanced the diagnostic performance of multiplexed biomarker tests. This study generated data in support of digital multiplexed strategies in digitally enabled settings for at-risk populations nationally and worldwide.

## Introduction

### Background

In Canada, at-risk populations are disproportionately impacted by sexually transmitted blood-borne infections (STBBIs). These populations include men who have sex with men; Indigenous, immigrant, and refugee populations; and people who inject drugs, among others [1-3]. The standard of care with regard to STBBI diagnostics entails laboratory-based testing, sometimes requiring invasive specimen collection, and providing results at a subsequent clinic visit several days later based on geographical location. Location and availability of tests impact the turnaround time to obtain test results, which affects timely counseling and initiation of treatment and care [4,5].

### Challenges in STBBI Diagnostics and Care for At-Risk Populations

Following the COVID-19 pandemic and the provision of self-tests that led to an improved understanding of testing and screening services, at-risk populations desire convenient and timely access to health care services for STBBIs, including demands for rapid testing, confirmation, counselling, and initiation of treatment in 1 to 2 visits, if possible [6]. However, the current status quo, with health care worker shortages and demands on professional time, limits patient access to sought services, namely due to the lack of dedicated family physicians, translating to many missed opportunities in STBBI screening [7-9].

In addition, marginalization of at-risk underserved populations, together with stigma, discrimination, and systemic racism, impacts health care seeking and timely screening for these important STBBIs despite availability of testing, further perpetuating health care inequity [10].

In parallel, in current times, in the context of acute health care professional shortages nationwide, populations demand the use of innovative patient-centered strategies that improve efficiency and costs of service delivery [11-13]. Mobile, rapid, connected digital screening programs fill health service delivery gaps in STBBI care, including efficient service delivery, data documentation, and linkage arrangement when screened using point-of-care (POC) technology-based services [14].

**Innovative Strategies for Improving STBBI Care**

As mentioned in the Lancet Commission on Diagnostics, digitization can improve access to screening and linkage and retention pathways for underserved populations [15]. Together with increasing access to timely diagnostics, the commission has called for an investment in the training of health care professionals using innovative solutions. The proposed solution is an investment to fill gaps in training, data documentation, communication, and linkages using POC technologies that will improve service delivery. To reach global targets, evidence to support the use of innovative and smarter connected screening solutions across different contexts and populations is much needed, for example, a study in a high-income context [6].

Since 2009, our laboratory has been at the forefront of creating digital process innovations in this space of POC diagnostics. We have developed screening digital solutions for HIV and hepatitis C virus (HCV) self-testing, multiplexed screening for STBBIs, COVID-19 self-testing, and sexual and reproductive health services. Innovative digital strategies can not only improve rapid STBBI screening and linkage of testers but also help in training, counselling, and data management by health care workers while optimizing the screening pathway. Digital strategies also address the gaps in data and increase efficiency by streamlining workflows thus enabling task shifting, all while providing data to guide health care service delivery [16].

The AideSmart! app- and platform-based solution integrated with multiplexed POC technologies, called the AideSmart! multiplexed strategy, is designed for health aides (frontline health care workers) and is one such unique solution.

The AideSmart! strategy was first pilot-tested in India in 2018 in pregnant women in a Grand Challenges Canada–funded study. In that study, the strategy was deemed successful in detecting 239 new infections or conditions (HIV, hepatitis B or C, and syphilis) and helped in reducing vertical transmission to infants [17]. While the pilot study showed the effectiveness of the AideSmart! strategy in rural India, a middle-income country, we wished to examine its feasibility in Canada, a high-income country with vastly different at-risk subpopulations with varying prevalence of STBBIs.

In this Canadian Institutes of Health Research–funded study, we adapted the innovative solution to meet the needs of Canadian vulnerable populations (people who inject drugs; sexually transmitted disease [STD] clinic attendees; lesbian, gay, bisexual, transgender, and queer individuals; and refugee and immigrant populations). We localized the digital platform into French, tailored the content to align with Canadian guidelines, and integrated the newest versions of multiplexed assays to offer a biomarker-based strategy to populations.

With a focus on global portability and uptake and the aim of introducing scalable solutions that address data and service delivery gaps using POC technologies for STBBI screening, counselling, and linkage to care, we sought to evaluate AideSmart! within a high-income setting using multiplexed strategies that are slowly changing the landscape of delivery of STBBI diagnostics at the POC across global settings.

## Methods

### Study Design and Objectives

In this cross-sectional study, our primary aim was to evaluate the feasibility, preference, and acceptability of the AideSmart! strategy among at-risk populations in Canada. Our secondary aim was to estimate the accuracy of the 2 investigational rapid multiplexed devices and demonstrate their impact on detection of new infections.

### Ethical Considerations

Ethics approval was obtained from the Research Institute of the McGill University Health Centre (2020-6048) and from the Horizon Health Network Research Ethics Board at the Saint John Regional Hospital (2020-2963). An informed consent form (ICF) was presented to each participant before study entry in their preferred language (English or French). The ICF described the ability to withdraw consent or opt out during the study in the event that participants no longer wished to continue the study. Participants received financial compensation during the study of CAD $25 (US $18.51) for completing visits 1 and 2; an extra CAD $10 (US $7.41) was provided if a third visit was required and completed.

Furthermore, to ensure data privacy and security, all data recorded on the AideSmart! app were encrypted (3-layer encoded) and protected on a secure Health Insurance Portability and Accountability Act (HIPAA)–compliant server. A keyed access to the server was provided to the principal investigator of the study. A study ID was created to store data. It contained a site-specific code and a randomly generated participant ID. No names were revealed to the data analysts and the staff conducting the study at any point of the study. This anonymized, deidentified data protection process guaranteed participant confidentiality and has been successfully deployed in our national and global studies. The master list that linked names with study IDs was available only to the clinicians responsible for patient care and was safeguarded for the entire duration of the study.

### Study Population

Eligible recruited participants were adults with unknown serostatus for HIV, HCV, and syphilis, including but not limited to (1) people who inject drugs, (2) men who have sex with men, and (3) immigrant populations, in Quebec and New Brunswick. Individuals in treatment for any coinfection or with any urgent medical condition requiring hospitalization were excluded.

Participants were recruited from 2 community-based service delivery sites in Quebec and New Brunswick: RÉZO, Montreal, Quebec (May 2021 to February 2022), and the Centre for Research, Education, and Clinical Care of At-Risk Populations (RECAP), Saint John, New Brunswick (March 2021 to August 2022). Convenience sampling was deployed.

Recruitment was managed by the community-based organizations. Social media platforms (Facebook and Grindr) were used to spread the word about the study. Posters were placed in community clinics to increase awareness of the study, and flyers and handouts were printed that explained the purpose of the study (approved by the Research Ethics Board) and shared with clinic attendees and in local magazines. Health care staff participated in community outreach events in the area to increase word of mouth regarding the study, leveraging their existing community-based networks. Enrolled participants were offered to partake in a “refer a friend” program to invite additional persons to participate. Existing relationships with communities were thus leveraged to increase recruitment.

RÉZO is a community organization active since 1991 that offers gay and bisexual cis- and transgender men various free health and wellness promotion programs. RÉZO provides HIV or HCV and STBBI prevention and testing [18].

RECAP is a community-based harm reduction clinic dedicated to improving prevention, diagnosis, and treatment of HCV in at-risk populations, many with substance use disorders. This nonprofit organization, active since 2014, provides opioid agonist therapy, harm reduction counseling, safe supplies, and clinic- and outreach-based screening for STBBIs, among other services [19].

### AideSmart! STBBI Rapid Multiplexed Screening Strategy

**Figure 1:**
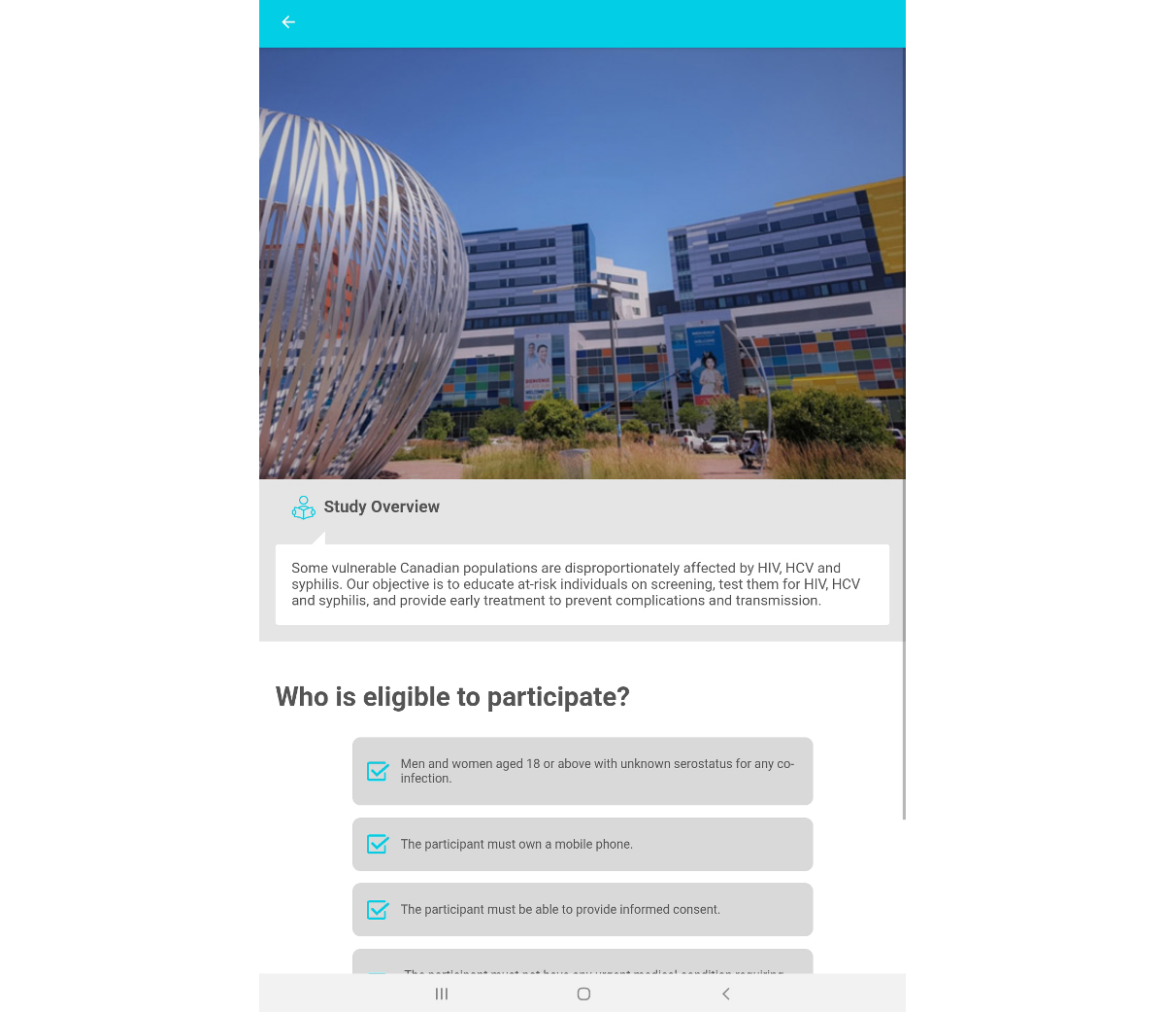
Screenshot of the AideSmart! app overview page.

**Figure 2:**
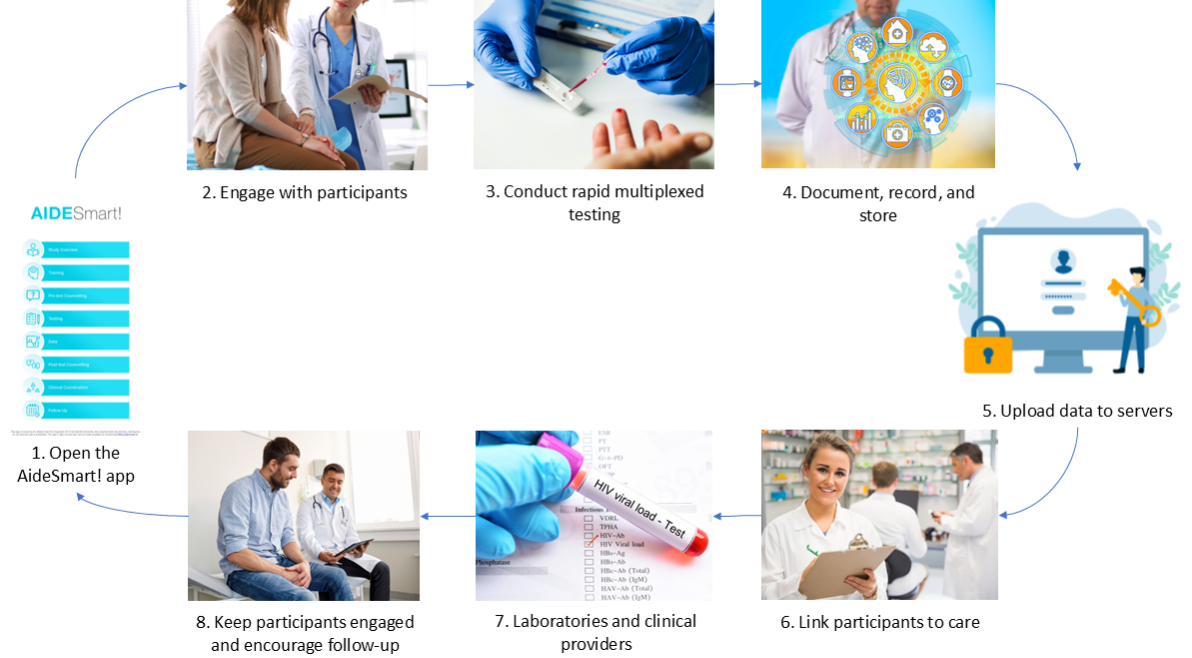
AideSmart! rapid multiplexed testing strategy to simultaneously screen for populations at risk of contracting sexually transmitted blood-borne infections in Canada.

The user-friendly AideSmart! app’s main page directs health aides to several tabs offering training on multiplexed tests, counselling, and recommendations for all STBBIs for both health care workers and testers. The Overview tab is shown in Figure 1, which presents the overall aim of the AideSmart! app and participant eligibility for study inclusion. The app also provides education and screening and testing information related to rapid multiplexed tests and arranges for communication with patients and health care providers while arranging for rapid linkage to confirmatory testing, treatment, and clinical care. As shown in Figure 2, the AideSmart! app was designed to streamline and simplify STBBI diagnostic care, document and store digital data, and link participants to care, all while maintaining high patient engagement and communication with various stakeholders at all times. The app- or web-based strategy is open access, portable, and customizable to both technologies: multiplexed platforms and rapid biomarker-based multiplexed assays. It is currently being adapted and tested using multiplexed molecular platforms in an India-Canada Centre for Innovative Multidisciplinary Partnerships to Accelerate Community Transformation and Sustainability–funded study in India.

The AideSmart! platform and app solution (open access, McGill University; copyright Report of Invention 16126, 2016) was integrated with 2 investigational lateral flow assays: Multiplo Rapid *Treponema pallidum* Antibody Test (MedMira Inc) and Dual Path Platform HIV-syphilis assay (Chembio Diagnostic Systems, Inc) [17]. Of note, the Chembio test for HIV or syphilis was operated using a quantitative reader that removed ambiguity associated with a visual test result interpretation, whereas the MedMira test relied on visual interpretation of test results.

In this study, these tests were used to independently evaluate accuracy and evaluate some prototypes of the rapid tests. Both rapid index tests required health care professional–collected finger stick blood–based samples. Confirmatory testing was performed using both the local laboratory and dried blood spot (DBS) tests as reference standard tests on each participant. Local laboratory testing per standardized algorithms (Textbox 1) was conducted in Quebec and New Brunswick. DBS specimens were sent for analysis at the National Laboratory for HIV Reference Services in Winnipeg, Manitoba.

The AideSmart! app-based program connected stakeholders throughout the study. The digital app-based platform detailed study procedures, training on rapid testing procedures, pretest and posttest combined counseling for STBBIs, clinical care and coordination for those who receive a positive test result, treatment, and follow-up. For counseling and treatment, Canada-specific guidelines for STBBIs were included in the app-based program to provide targeted information to participants [20].

**Local laboratory screening algorithms in Canada for HIV, syphilis, and hepatitis C virus (HCV).**

**HIV**

- Geenius HIV 1/2 test (Quebec) or Architect HIV 1/2 Ag/Ab Combo assay (New Brunswick)
- If reactive: confirmatory p24 enzyme immunoassay (EIA)
- If reactive: specimen sent to the National Microbiology Laboratory for final analysis/confirmation

**Syphilis**

- Enzyme immunoassay (EIA)
- If reactive: Rapid plasma regain (RPR) test
- If needed, *Treponema pallidum* particle agglutination (TP-PA) and INNO-LIA tests

**HCV**

- Anti-HCV screen
- If reactive: qualitative RNA test
- If reactive, viral load measured
- Note: If either test yielded non-reactive results but the test was conducted in the window period following suspected HCV exposure, follow-up test was recommended three months later

### Study Procedures and Intervention

We recruited throughout the COVID-19 pandemic, and communication was facilitated through SMS text messages and calls enabled by our AideSmart! app strategy.

At visit 1, following an explanation of the study objectives and study procedures by the health care professionals, consenting participants were enrolled in the study. Using the AideSmart! innovation, health care professionals conducted pretest counseling aiming to educate participants regarding STBBIs, rapid and laboratory-based testing methods, and posttest linkage and treatment options for each STBBI.

Participants had the opportunity to ask questions as needed. Next, rapid MedMira and Chembio tests were performed per manufacturer instructions. As both MedMira and Chembio were considered investigational devices (not approved in Canada yet), test results were declared following receipt of local laboratory test results, although turnaround time to obtain rapid test results was recorded in real time. Health care professionals documented deidentified data on participants’ demographics, risk profile, and rapid test results.

Between visits 1 and 2, health care professionals and participants communicated using participants’ preferred method of communication. Health care professionals sent SMS text message reminders to confirm the date and time of the follow-up appointment, also allowing for sufficient time for confirmatory test results to be expedited from local laboratories. All participants returned for a second visit, during which both positive and negative test results were declared in a posttest counseling or harm reduction session aided by the AideSmart! app. Health care professionals documented rapid and confirmatory test completion and preference data. Participants with nonreactive test results were encouraged to seek repeat testing should future exposure be expected. In case of one or more reactive result or results, linkage to treatment was arranged, either in-house or at a nearby clinic.

For follow-up and retention in visit 3, health care professionals coordinated another visit for those who received a positive test result.

### Feasibility

Feasibility was computed using the metric completion rate, defined as the number of participants who successfully completed the procedure that consisted of pretest counselling and testing (visit 1) and posttest counselling and linkage to treatment initiation (visit 2; numerator) divided by the total number of participants who consented to take part at baseline (denominator).

The primary end point was used for sample size calculation. On the basis of our previous evaluations, with an assumed completion rate of 70%, a sample size of 323 was deemed sufficient with 95% level of confidence and 5% precision [21].

Acceptability was defined as the number of participants who consented to the strategy (numerator) over the number of eligible participants who were approached (denominator).

### Preference

Preference was defined as the number of participants who indicated a preference for a patient-reported outcome (including preference of timing to receive test results and retest preference) divided by the number of participants who consented to take part in the study (denominator). Satisfaction of POC and conventional testing was further recorded categorically (*very satisfied*, *satisfied*, *neutral*, *dissatisfied*, and *very dissatisfied*).

### Health Care Provider Perspectives

Health care professional feedback was collected to gain perspective regarding their views on multiplexed rapid testing. The following questions were asked: “What do you think about multiplex tests?” “Do you think they will have a future in Canada?” “If approved, do you think they will be useful for populations they serve?” “Is it helpful for people to know if they test negative?”

### Impact

Impact was defined as the number of newly diagnosed infections (numerator) relative to the number of patients who consented and were screened (denominator).

Impact was also recorded using the metric that captures turnaround time, defined as the mean time recorded in minutes or days for test results to be obtained so as to inform the next step in the screening pathway.

### Diagnostic Accuracy

Diagnostic accuracy performance metrics (sensitivity, specificity, and 95% CIs) for each pathogen were tabulated for index tests (Chembio and MedMira) separately and compared to both reference standard tests (local laboratory algorithms and DBS).

Participants’ sociodemographic profiles were tabulated.

Outcome data (feasibility, acceptability, preference, and impact) were calculated in proportions with 95% CIs. All analyses were performed in Stata (version 10; StataCorp) [22] and R (version 4.1.3; R Foundation for Statistical Computing) [23].

## Results

### Study Population

In total, we recruited 401 participants (n=237, 59.1% from RÉZO and n=164, 40.9% from RECAP).

Overall, 71.6% (282/394) of participants were men, 21.6% (85/394) were women, and 6.9% (27/394) identified as another gender. In terms of ethnicity, 5.6% (22/394) self-reported being Indigenous individuals, 5.8% (23/394) self-reported being Asian or Mediterranean individuals, 9.6% (38/394) self-reported being Latin American or Caribbean individuals, 5.3% (21/394) self-reported being African individuals, and 73.6% (290/394) self-reported being White individuals.

Most participants (165/394, 41.6%) were young (aged 18-34 years), 59.4% (234/394) were doing part-time work or were unemployed, and 65.5% (258/394) earned <CAD $2000 (US $1480.26) per month.

Although 86% (339/394) of the participants had completed high school or advanced degrees, nearly half (172/394, 43.7%) of the participants were unemployed (Table 1).

In the previous year, with respect to past testing history, nearly half of the participants had been tested for HIV (184/392, 46.9%), HCV (185/392, 47.2%), and syphilis (172/391, 43.9%). A subset of participants (169/385, 43.9%) reported a previous STD, the most common being chlamydia (72/169, 42.6%), syphilis (48/169, 28.4%), and gonorrhea (47/169, 27.8%).

Regarding risk profile, while most (288/394, 73.1%) reported being sexually active, many (247/394, 62.7%) reported no stable partners. Approximately half (159/379, 42%) reported 2 to 5 sexual partners in the previous 6 months, and half (167/370, 45.1%) reported occasional condom use. Approximately half had never been diagnosed with a previous STD (204/385, 53%), had never injected recreational drugs (250/392, 63.8%), or were not alcohol users (206/393, 52.4%). Among people who injected drugs, most (83/139, 59.7%) had shared needles in the past.

Men presented with a riskier baseline status. Of the 60 participants with at least 6 recent sexual partners, 47 (78%) were men, whereas 13 (22%) were women or identified as another gender. Moreover, a higher proportion of men (122/272, 44.9%) reported a previous STD compared to women and those who identified as another gender (38/112, 33.9%).

In addition, the 43.9% (169/385) of participants who had a previous STD presented with riskier baseline sexual behaviors. For instance, 20.7% (35/169), 49.1% (83/169), and 20.7% (35/169) reported always, sometimes, and never using a condom, respectively. Moreover, 73.4% (124/169) had 0 to 5 recent sexual partners, and 24.9% (42/169) had ≥6 partners. Conversely, participants with no previous recorded STDs (216/385, 56.1%) had less risky behaviors, including a higher rate of protected sex (67/216, 31%) and up to 5 recent sexual partners (191/216, 88.4%). However, participants with a previous STD seemed more aware of this riskier behavior, as indicated by higher previous testing rates (14%-19% vs 7%-8%).

**Table 1:** Demographics and medical history of recruited study populations to the AideSmart! cross-sectional multiplexed rapid sexually transmitted blood-borne infection screening study in Canada (N=394).

Criteria			Participants, n (%)
**Age (y)**			
	18-24	32 (8.1)	
	25-34	133 (33.8)	
	35-44	112 (28.4)	
	45-54	63 (16)	
	≥55	54 (13.7)	
**Gender**			
	Man	282 (71.6)	
	Woman	85 (21.6)	
	Another gender	16 (4.1)	
	No response	11 (2.8)	
**Descent**			
	African	21 (5.3)	
	Asian or South Asian	10 (2.5)	
	European or North American	290 (73.6)	
	Latin American or Caribbean	38 (9.6)	
	Mediterranean	13 (3.3)	
	North American Indigenous	22 (5.6)	
**Educational level**			
	Did not complete primary schooling	55 (14)	
	High school to preuniversity education	163 (41.4)	
	Technical degree	55 (14)	
	Postgraduate degree	121 (30.7)	
**Employment status**			
	Employed full time	130 (33)	
	Employed part time	62 (15.7)	
	Not employed	172 (43.7)	
	Retired	11 (2.8)	
	Not willing to work	19 (4.8)	
**Monthly income**			
	<CAD $2000 (US $1480.26)	258 (65.5)	
	CAD $2001-$4000 (US $1481-$2960.52)	105 (26.6)	
	CAD $4001-$6000 (US $2961.26-$4440.78)	22 (5.6)	
	CAD $6001-$8000 (US $4441.52-$5921.04)	7 (1.8)	
	≥CAD $8001 (US $5921.78)	2 (0.5)	
**Currently partnered**			
	Yes	147 (37.3)	
	No	247 (62.7)	
**Sexual activity**			
	Yes	288 (73.1)	
	No	88 (22.3)	
	I do not wish to answer	18 (4.6)	
**Condom use (n=370)**			
	Never	98 (26.5)	
	Always	105 (28.4)	
	Sometimes	167 (45.1)	
**Recent sexual partners (past 6 months; n=379)**			
	0	66 (17.4)	
	1	94 (24.8)	
	2-5	159 (42)	
	6-10	38 (10)	
	≥11	22 (5.8)	
**Previous STDs^a^ (n=385)**			
	Yes	169 (43.9)	
	No	204 (53)	
	I do not know	12 (3.1)	
**Type of previous STD^b^ (n=169)**			
	HIV	10 (6)	
	Herpes simplex	3 (1.8)	
	Chlamydia	72 (43.1)	
	Gonorrhea	47 (28.1)	
	Syphilis	48 (28.4)	
	Other	25 (15)	
**Previous recreational injection drug use (n=392)**			
	Yes	139 (35.5)	
	No	250 (63.8)	
	I do not wish to answer	3 (0.8)	
**If yes to previous recreational injection drug use, were needles shared? (n=139)**			
	Yes	83 (59.7)	
	No	56 (40.3)	
**Alcohol use (n=393)**			
	No	206 (52.4)	
	1-2 times per week	122 (31)	
	3-5 times per week	65 (16.5)	
**Previous HIV test (n=392)**			
	No	54 (13.8)	
	Yes, <6 months ago	137 (34.9)	
	Yes, 6 months to 1 year ago	47 (12)	
	Yes, >1 year ago	154 (39.3)	
**Previous HCV** ^c^ **test (n=392)**			
	No	41 (10.5)	
	Yes, <6 months ago	137 (34.9)	
	Yes, 6 months to 1 year ago	48 (12.2)	
	Yes, more than 1 year ago	166 (42.3)	
**Previous syphilis test (n=391)**			
	Yes, <6 months ago	125 (32)	
	Yes, 6 months to 1 year ago	47 (12)	
	Yes, more than 1 year ago	219 (56)	

^a^STD: sexually transmitted disease.

^b^Patients may have reported >1 previous STD.

^c^HCV: hepatitis C virus.

### Feasibility

All participants approached (401/401, 100%) accepted the AideSmart! strategy. Completion of AideSmart! over 2 visits was 76.1% (305/401).

The mean turnaround time to receive multiplexed rapid test results was <15 minutes (mean 13.9, SD 2.1 min for Chembio and mean 13.5, SD 2.3 min for MedMira). In comparison, mean turnaround time for local laboratory test results ranged from 4 days to 3 weeks (HIV p24, mean 4.4, SD 3.0 days; HCV RNA, mean 9.0, SD 6.2 days; HIV RNA, mean 11.2, SD 5.1 days; *T pallidum* particle agglutination, mean 12.4, SD 6.5 days; DBS, mean 24.2, SD 5.8 days).

### Preference

Nearly all participants (243/247, 98.4%) reported high or very high satisfaction with the rapid testing and counseling through the AideSmart! strategy versus 95.8% (226/236) who reported satisfaction with the conventional laboratory-based strategy. Participants reported comparable fear, pain, and discomfort between the rapid and conventional strategies (Table 2).

Preference to obtain same-day test results using rapid testing was expressed by 56.6% (141/249) of the participants. Preference for follow-up through various digital health supports varied—the most popular response was app-based secure messaging (84/250, 33.6%), followed by face-to-face visits (60/250, 24%), phone calls (39/250, 15.6%), and SMS text messaging (34/250, 13.6%); 13.2% (33/250) had no preference. As a result, unsurprisingly, 93.6% (234/250) of the participants reported that they would recommend rapid testing to friends.

**Table 2:** Acceptability, feasibility, and preference results of the AideSmart! cross-sectional multiplexed rapid sexually transmitted blood-borne infection screening study in Canada (2021-2022).

Criteria				Participants, n (%)
**Acceptability (n=401)**				
	Yes			401 (100)
**Completion of pretest counseling, testing, posttest counseling, and linkage to treatment (N=401)**				
	Yes			305 (76.1)
	No			96 (23.9)
**Point-of-care testing**				
	**Fear^a^** **(n=249)**			
		No fear (1)	161 (64.7)	
		2	53 (21.3)	
		3	19 (7.6)	
		4	9 (3.6)	
		Highest fear (5)	7 (2.8)	
	**Pain^b^** **(n=248)**			
		No pain (1)	171 (69)	
		2	61 (24.6)	
		3	15 (6)	
		4	1 (0.4)	
	**Discomfort^c^** **(n=248)**			
		No discomfort (1)	185 (74.6)	
		2	49 (19.8)	
		3	11 (4.4)	
		4	2 (0.8)	
		Highest discomfort (5)	1 (0.4)	
	**Satisfaction (n=247)**			
		Very satisfied	213 (86.2)	
		Satisfied	30 (12.1)	
		Neutral or not sure	4 (1.6)	
**Conventional testing**				
	**Fear^a^** **(n=235)**			
		No fear (1)	152 (64.7)	
		2	46 (19.6)	
		3	22 (9.4)	
		4	10 (4.3)	
		Highest fear (5)	5 (2.1)	
	**Pain^b^** **(n=236)**			
		No pain (1)	162 (68.6)	
		2	62 (26.3)	
		3	11 (4.7)	
		4	1 (0.4)	
	**Discomfort^c^** **(n=236)**			
		No discomfort (1)	175 (74.2)	
		2	44 (18.6)	
		3	14 (5.9)	
		4	2 (0.8)	
		Highest discomfort (5)	1 (0.4)	
	**Satisfaction (n=236)**			
		Very satisfied	192 (81.4)	
		Satisfied	34 (14.4)	
		Neutral or not sure	9 (3.8)	
		Dissatisfied	1 (0.4)	
**Preference of timing to receive test results (n=249)**				
	1 day			141 (56.6)
	<1 week			99 (39.8)
	<2 weeks			7 (2.8)
	≥2 weeks			2 (0.8)
**Retest preference (n=249)**				
	Rapid test			148 (59.4)
	Phlebotomy			67 (26.9)
	No preference			34 (13.7)
**Rapid test recommendation to friends (n=250)**				
	No			7 (2.8)
	Yes			234 (93.6)
	Not sure			9 (3.6)
**Preference for follow-up (n=250)**				
	Phone call			39 (15.6)
	SMS text messaging			34 (13.6)
	App-based secure messaging			84 (33.6)
	Face-to-face			60 (24)
	No preference			33 (13.2)

^a^Mean score for point-of-care: 1.6 (SD 1.0); conventional: 1.6 (SD 1.0).

^b^Mean score for point-of-care: 1.4 (SD 0.6); conventional: 1.4 (SD 0.6).

^c^Mean score for point-of-care: 1.3 (SD 0.6); conventional: 1.3 (SD 0.7).

### Health Care Provider Perspectives

Health care professionals were questioned, and their perspectives were tabulated qualitatively (Textbox 2). Across all queries, favorable responses were conveyed. For instance, health care professionals deemed multiplexed tests to be user-friendly and accurate when compared to reference standard tests. The overall perspective was that they would add value in Canada if implemented in routine care in community settings, although it was noted that acquiring a proper patient medical history was important to identify new infections. The rapid multiplexed test use was considered useful whether the test result was negative or positive; a negative status provided multiple benefits as patients’ anxiety would diminish and the result would serve as a baseline for subsequent testing.

**Health care provider perspective of multiplexed tests in the context of the AideSmart! cross-sectional multiplexed rapid sexually transmitted blood-borne infection screening study in Canada (2021-2022).**

**What do you think about multiplexed tests?**

- “I really like the multiplex tests. They are super user friendly and I think they have immense potential for providing comprehensive screening to vulnerable populations.”
- “They should always be paired with such a positive blood test.”
- “I enjoy using multiplex tests, they are simple to use and provide quick results. I also have found that the results have been consistent with bloodwork results, or dried blood spots.”

**Do you think they will have a future in Canada?**

- “I think they have huge potential in Canada. I can see them being used in the community setting to provide on the spot screening.”
- “Yes, but it’s important to be careful for customers with one of the diagnoses already known.”
- “I believe that they will have a future in Canada, our clinic has been using point of care testing for HCV screening before the multiplex study, and I believe the multiplex tests are an improvement as we can test for two/three bloodborne pathogens as opposed to one.”
- “It will take some time to get approved.”

**If approved, do you think they will be useful for the populations they serve?**

- “In the population I work with it can be challenging to collect bloodwork, and patients are hesitant to have it done. With the multiplex test it requires minimal blood, and is noninvasive, which makes the patients more likely to participate.”
- “If approved the multiplex test will be useful in our population, as it is important for patients to have results on site.”

**Is it helpful for people to know if they test negative?**

- “This is the reason why they take the test; people want to know if they are not contagious.”
- “It provides relief and ease of mind to the patient. Furthermore, it serves as a baseline for rescreening.”
- “I think knowing a negative status is just as important as knowing a positive status.”

### Impact

With respect to new infections, 29 new infections (n=27, 93% HCV; n=1, 3% HIV; and n=1, 3% syphilis) were detected using the AideSmart! multiplexed strategy in at-risk populations. Of the 29 new infections, 8 (28%) spontaneously cleared and required no further follow-up. Among the remaining 21 infected participants, 12 (57%) received follow-up treatment and care in visit 3, a total of 2 (10%) died, 4 (19%) were incarcerated, and 3 (14%) were completely lost to follow-up. Furthermore, 94.7% (380/401) of the participants did not require a visit 3 follow-up (participants who tested negative for all 3 pathogens, 372/380, 97.9%; and those who tested positive for HCV but the virus spontaneously cleared and they required no treatment, 8/380, 2.1%).

While the study recruitment occurred during the COVID-19 pandemic, with this strategy, we were able to track 99.3% (398/401) of the participants throughout the study. The pandemic-induced restrictions made it difficult for us to keep participants in care; despite the attempts made by the staff to retain these patients, loss to follow-up was inevitable.

Of note, 38% (11/29) of participants who tested positive had had a previous STD, and 38% (11/29) reported never using condoms at baseline. Interestingly, with the exception of 14% (4/29) of the participants, who were not comfortable disclosing their past sexual history, all individuals with new infections (25/29, 86%) had 0 to 5 recent sexual partners, thereby indicating that even persons with stable or few partners are at risk of contracting a sexually transmitted infection (STI). Moreover, most participants with new infections were unemployed (22/29, 76%), identified as a man (21/29, 72%), and aged ≥35 years (20/29, 69%).

### Diagnostic Accuracy

The diagnostic accuracy of both rapid tests is presented in Tables 3 and 4, compared to reference standard tests through local laboratory assays in Table 3 and DBS in Table 4.

For HIV, both MedMira and Chembio reported a high sensitivity and specificity (>98%) when compared to local laboratory tests as a reference standard. With DBS as a reference standard alone, the sensitivity of the MedMira test for HIV was 93.8% (95% CI 69.8%-99.8%).

For HCV, the MedMira test was the sole rapid test that detected HCV antibodies; it reported a high sensitivity at 91.2% (95% CI 84.3%-95.7%) and a high specificity at >99% (95% CI 97.5%-99.9%).

For syphilis, against local laboratory tests, for both rapid tests, specificity was high (>99%). However, the sensitivity of the Chembio test varied (relative to local laboratory tests: 86.8%, 95% CI 71.9%-95.6%; relative to DBS: 80%, 95% CI 64.4%-91%). MedMira’s sensitivity varied as well (relative to local laboratory tests: 57.9%, 95% CI 40.8%-73.7%; relative to DBS: 55%, 95% CI 38.5%-70.7%). This statement holds true while comparing test results between the rapid tests and both reference standard tests.

**Table 3:** Diagnostic accuracy of the Chembio and MedMira point-of-care (POC) tests in comparison to local laboratory tests in the AideSmart! cross-sectional multiplexed rapid sexually transmitted blood-borne infection screening study in Canada (2021-2022).

POC test		Sensitivity, % (95% CI)	Specificity, % (95% CI)
**HIV**			
	Chembio	100.00 (79.41-100.00)	98.71 (96.72-99.65)
	MedMira	100.00 (78.20-100.00)	100.00 (98.82-100.00)
**Syphilis**			
	Chembio	86.84 (71.91-95.59)	99.65 (98.06-99.99)
	MedMira	57.89 (40.82-73.69)	100.00 (98.72-100.00)
**Hepatitis C**			
	Chembio	—^a^	—
	MedMira	90.32 (80.12-96.37)	99.62 (97.92-99.99)

^a^Not applicable.

**Table 4:** Diagnostic accuracy of the Chembio and MedMira point-of-care (POC) tests in comparison to dried blood spot tests in the AideSmart! cross-sectional multiplexed rapid sexually transmitted blood-borne infection screening study in Canada (2021-2022).

POC test		Sensitivity, % (95% CI)	Specificity, % (95% CI)
**HIV**			
	Chembio	100.00 (76.84-100.00)	98.94 (97.30-99.71)
	MedMira	93.75 (69.77-99.84)	100.00 (99.02-100.00)
**Syphilis**			
	Chembio	80.00 (64.35-90.95)	99.03 (97.19-99.80)
	MedMira	55.00 (38.49-70.74)	100.00 (98.81-100.00)
**Hepatitis C**			
	Chembio	—^a^	—
	MedMira	91.15 (84.33-95.67)	99.29 (97.46-99.91)

^a^Not applicable.

## Discussion

### Principal Findings

In this study, we report high acceptability (401/401, 100%), satisfaction (393/401, 98%), and feasibility (305/401, 76.1%) for the AideSmart! multiplexed strategy. Participants preferred multiplexed rapid testing (148/249, 59.4%) over conventional laboratory-based testing, primarily due to its ability to provide a test result (negative or positive) in a shorter turnaround time.

Study findings demonstrate the strategy’s relevance for Canadian at-risk populations, as illustrated by the importance of obtaining a negative test result in at-risk populations and its overall impact in detecting new infections. With respect to impact, at the RECAP site, we detected most new infections (26/29, 90%), all of which were HCV, whereas at RÉZO, we detected 3 new infections (n=1, 33% HIV; n=1, 33% HCV; and n=1, 33% syphilis). Furthermore, all participants obtained laboratory-confirmed test results, were engaged throughout the process, stayed in communication with health care aides regarding their test results, and were counselled in time. Furthermore, treatment decisions were expedited in 3 visits for participants who tested positive. Most participants (377/401, 94%) did not require treatment as many infections spontaneously resolved. Although many participants who required follow-up (12/21, 57%) were linked and retained in care in visit 3, due to COVID-19 restrictions and repeat lockdowns, tracking 43% (9/21) of the participants was difficult. However, we were notified that 22% (2/9) died and 44% (4/9) were incarcerated. Despite the best efforts of our staff, 33% (3/9) left the province or were lost to follow-up. Overall, using this digital strategy, 99.3% (398/401) of our participants were tracked throughout the pandemic.

To the health care providers, knowledge of serostatus for STBBIs with rapid tests allowed for planning of screening and confirmatory testing and counselling for these participants at a time during the pandemic when services were interrupted for many STBBIs. This positive impact can be further enhanced by a rapid turnaround time of 15 minutes (once approved and implemented) for preliminary test results, as opposed to a waiting time of 4 to 24 days to obtain laboratory-based confirmatory results. The long waiting time carries the possibility of losing at-risk populations.

Health care providers underscored the importance of declaring both positive and negative test results using multiplexed rapid test results. Indeed, as the study population comprised at-risk individuals, the knowledge of one’s negative status is crucial for infection control. Although false positive results are troublesome, false negative results can lead to more devastating outcomes when used for screening, including a false sense of security and higher likelihood of engaging in risky behaviors fueling forward transmission. Health care providers expressed positive feedback regarding the multiplexed testing strategy and were convinced of their use in future screening initiatives. The digital strategy allowed for the tracking of participants’ care throughout the care pathway and maximized engagement of health care workers and participants during the COVID-19 pandemic, especially when health care professionals knew and used patients’ preferred method of communication.

One very encouraging finding was the consistently high specificity of the rapid tests for the pathogens, often approaching 99% to 100% with biomarker-based tests, suggesting that both tests can be used with confidence to rule out disease. This property was noted by our health care providers. As such, negative test results could be considered negative unless proven otherwise through a second confirmatory laboratory test. This saves time and energy for health care systems and allows for task shifting and for focus to shift to new infections based on individuals’ history and presentation. This was relevant during the pandemic, when the possibility of bringing underserved populations to screen for infections, in the context of health care staff shortages was very low.

This study is the first in Canada to assess the diagnostic accuracy of the multiplexed Chembio Dual Path Platform assay (with an electronic reader) and of MedMira’s investigational Multiplo Rapid *T pallidum*, HIV, and HCV Antibody Test in Canada and the first to demonstrate the success of a digital connected strategy for multiplexed biomarker tests for triple infections. We observed that, to increase the accuracy (sensitivity) of detection, electronic readers were extremely useful. They helped reduce the ambiguity faced in field implementation with respect to rapid test result interpretation; this has been noted in other studies or sites. With electronic readers, health care professionals could state with more confidence that observed positive results were true positives and that observed negative results were true negatives. This was observed with the syphilis and HIV Chembio test results in this study. Rapid tests, if used in combination with electronic readers, will be extremely useful for expanded access and screening initiatives in outreach and community-based settings where laboratory-based tests are not readily available in a rapid time and where the endemicity of these infections in subpopulations is high and the chance of losing a tester to follow-up is also high. An important strength of the MedMira test is the ability to accurately screen for HCV using a blood sample in a short span of time. However, the Chembio test demonstrated an improved and increased sensitivity for HIV and syphilis with the quantitative reader, increasing confidence in the test result. MedMira fell short, particularly in screening for syphilis; the Chembio test performed very well with an electronic reader. As both are lateral flow assays, a visual assessment is required to interpret findings. However, the use of an electronic reader with the Chembio test improves the health care professional’s task and reduces incorrect interpretation. With many rapid assays without electronic readers, faint test lines or dots are missed, impacting sensitivity estimations.

Last year, global diagnostic organizations such as the Foundation for Innovative New Diagnostics (FIND), Geneva [24], and the World Health Organization (WHO) released their new guidelines. These guidelines have called for a greater use of electronic readers integrated with POC tests, apps, and digital supports that can enhance an accurate reading of rapid test results—an attribute that was noted as an advantage with connected rapid biomarker-based tests [25,26].

Diagnostic accuracy with biomarker-based assays varies between devices, for pathogens, against different reference standard tests, and for populations with varying prevalence of coinfections, as is the case with many rapid tests [5,27]. It is important to note additional factors that may impact the sensitivity of a multiplexed test. To corroborate this, in our previous studies with multiplexed tests without apps in India and Montreal, we observed that factors such as the immunogenicity of the patient, level of immune compromise, presence, prevalence, and endemicity of STBBIs, number of biomarkers present and type of device, and incidence of STBBIs in the population tested impacted the avidity that in turn affected the sensitivity of biomarker-based rapid tests [27]. In a few editorials, we have discussed the usefulness of these assays for subpopulations in national and global settings [5,27-29]. We found that 2-pathogen multiplexed biomarker rapid tests were easier to implement than a 3-pathogen test [30-33]. On the basis of our experience, we recommend the use of 2-pathogen rapid biomarker-based tests together with quantitative readers that help improve test interpretation capacity at the POC. As both rapid tests detected antibodies, several positive rapid test results were associated with treated previous infections.

Of note, the availability of medical history and risk profiles or electronic medical records on file and information collected using the AideSmart! app becomes especially important and is relevant as it helps make sense of a new diagnosis of infections or reinfections in outreach settings.

While no study to the best of our knowledge has presented the field-based diagnostic accuracy of a connected multiplexed strategy using biomarker tests, few HIV or syphilis rapid tests are commercially available. Similar to our results, their specificity is generally very high (>98%), but sensitivities vary [33]. For instance, the sensitivity of the bioLytical INSTI test is 56.8% for syphilis but is high (98.8%) for HIV [34]. The SD Bioline test yields an impressive sensitivity of 86.4% to 100% for syphilis and 99.1% to 100% for HIV [30-32,35]. However, the Biosynex Triplex HIV, HCV, and HBsAg test has reported an optimal (100%) diagnostic accuracy for all 3 pathogens [36]. We recommend the use of electronic readers for the tests reporting poor sensitivity to enhance their performance.

While multiplexed rapid biomarker-based tests offer a potential to screen for multiple pathogens in a short turnaround time, saving time and money to the health care system and the testers, they also catalyze the screening pathway for the populations who need multiplexed rapid biomarker-based tests through their availability and accuracy. To aid health care professionals in selecting the most appropriate test in their setting, we have published a systematic review that documents the accuracy of all rapid multiplexed assays and platforms [33]. While rapid lateral flow assay tests were used in this study, it is worth noting that platform devices can also yield rapid same-day test results. Multiplexed platform diagnostic technologies have been shown to be highly accurate using metrics of diagnostic performance (sensitivity and specificity). Certain devices yield such high diagnostic accuracy that they can be used to confirm infection [33], obviating the need to conduct or call for further laboratory-based tests. Portable platforms have the potential to transform the testing or treatment for these infections in community-based outreach settings; however, their need for laboratory infrastructure prevents their use in field settings. That said, the test results are available in 1 to 2 visits, and their potential to bring individuals to linkage if set up judiciously remains high. Such combination strategies of connected app-based rapid tests and portable platform or biomarker tests with electronic readers, if used together, can be useful to achieve elimination targets for HIV, HCV, and syphilis. Platform technologies detect nucleic acids as opposed to antibodies, and their cartridges remain expensive, but they will be more useful in diagnosing and treating new infections. However, these larger benchtop devices are associated with limited portability and high cost, and some require infrastructure such as air conditioning, which impedes their use in high temperature–prone outreach settings. While the benefits of platform devices may outweigh their drawbacks for some centers, biomarker-based flow-through multiplexed devices are cost-effective, affordable, and easy to use and represent an important and viable diagnostic option for populations with limited access to testing, at the very least to rule out infection given their high specificity.

### Study Strengths and Relevance

This study is the first to offer, evaluate, and generate evidence for the use of an integrated app-based multiplexed rapid STBBI testing strategy for at-risk Canadian populations, namely, people who inject drugs and immigrant and refugee populations. This study was conducted during and participants were recruited throughout the COVID-19 pandemic. For this study, the AideSmart! app was tailored to the Canadian context and test devices to enhance its global portability. This study represented key populations that typically need or desire rapid and regular STBBI screening. Participants were recruited from 2 sites, of which one specializes in HCV screening and care and the other provides services to immigrant and refugee populations. Our findings highlight that the AideSmart! multiplexed strategy impacted detection of new HCV, syphilis, and HIV infections with a rapid turnaround time; helped initiate counselling, linkage, and pre-exposure prophylaxis plans for those who tested both negative and positive; and helped track 99.3% (398/401) of our participants throughout the pandemic.

### Study Limitations

The study findings should be interpreted cautiously considering certain design limitations. Although our study design aimed to minimize biases, convenience sampling raises the potential for possible selection bias (sampling bias). Individuals willing to undergo STBBI testing may be more likely to be infected with an STBBI as these persons are more aware of their riskier lifestyle. This assumption was corroborated through our findings—study participants with a previous STD reported higher past testing rates when compared to participants without a previous STD. However, even persons with less risky lifestyles are at risk of contracting an STBBI, which illustrates the importance of increasing awareness among various populations to increase testing and, therefore, detection and treatment. We attempted to reduce misclassification bias by declaring results only upon receipt of laboratory-confirmed test results. Detection, ascertainment, and verification biases were minimized through an independent verification of test results from 3 laboratories.

As the study was entirely conducted during the COVID-19 pandemic, recruitment and retention presented many challenges, which led to delays in study completion. These were namely due to stay-at-home orders combined with individuals’ hesitancy to get tested during such uncertain times. Furthermore, while rapid test results were obtained in a few minutes, they could only be provided at visit 2 due to the investigational nature of the rapid tests and the need to wait for the test results from the laboratories, as well as the directives regarding clinic visits during the COVID-19 pandemic. The pandemic restrictions may have contributed to participants’ attrition at visit 2. However, it is worth noting that, in clinical care, the use of POC antibody-based tests limits the ability to provide definitive same-day positive screening results as further confirmatory testing is required. However, if confirmatory testing is expedited, treatment initiation is possible in visit 2, as was done in our study.

### Implications for Practice

Going one step further from rapid testing, the open access digital AideSmart! strategy is able to integrate multiplexed tests and platforms, thereby optimizing connected initiatives and saving health aide time in field or practice settings. Connected diagnostics and digitization represent an encouraging development during the COVID-19 pandemic and simplify notification of test results [15,37]. Connected solutions increase convenience and expedited access to screen, confirm, treat, and retain individuals in care, encouraging task shifting. This study illustrates the value and impact of an innovative, connected strategy for quality screening, patient engagement, data management, communication, linkages to care, and tracking of participants during the pandemic [38-40]. In addition to testing, the possibility to train health care professionals in each setting allows for quality control and quality assurance, which bodes well for certification and helps maintain a high quality of rapid POC tests, which is a concern in field implementation. Moreover, it allows for efficiency in data management and timely linkage to services.

The combination of accurate rapid tests and connected digital solutions fulfills several stakeholder needs. While individuals being tested generally seek highly accurate results with a quick turnaround time, the use of rapid tests together with a digital solution will significantly impact diagnostic care in several ways. Tasks are alleviated for health care professionals as the app-based program provides testing guidance, allows for result documentation, and improves stakeholder communication. Additional studies have shown the benefits of digital health in STBBI care. A scoping review by Cao et al [16] illustrated that digital platforms can improve STI prevention, accessibility and acceptability of testing, understanding and uptake of pre-exposure prophylaxis, surveillance, and postdiagnostic care (such as linkage and retention in care) for individuals who tested positive for at least one STI. This endeavor aligns with several global initiatives, such as those by the WHO [41] and the FIND [42], to integrate the use of digital health in health care services, including diagnostic care.

In terms of the generalizability of the findings, this study builds upon a previous AideSmart! study (Grand Challenges Canada funded) in India [17], a middle-income country, in a different at-risk population: pregnant women who were tested for bacterial and viral STBBIs. With a Canadian Institutes of Health Research–funded Canadian evaluation, we can confidently state that this rapid multiplexed screening strategy could be applied to populations desiring STBBI screening in high- and middle-income settings, including people who inject drugs; pregnant women; STD clinic attendees; and immigrant, refugee, and gender diverse populations. With new funding from the National Institutes of Health, in the coming years, we will aim to expand this program for multiple domains and infections and adopt it for use among health care workers in India [43], Uganda, and Ecuador [44].

Moreover, in Canada, while there is currently only 1 approved multiplexed test (INSTI) that is commercially available, it has a sensitivity of 98.85% (95% CI 93.4%-100%) and a specificity of 100% (95% CI 98.1%-100%) for HIV but a low sensitivity of 56.8% (95% CI 44.7%-68.2%) and a specificity of 98.5% (95% CI 95.7%-99.7%) for syphilis [34]. There is an imperative need to approve other high-performing tests such as Chembio (for syphilis and HIV) and Multiplo (for HCV and HIV) to meet the demand for multiplexed tests but also to drive down the prices of rapid tests. These tests can help expand the offer to screen at-risk Canadians, who regularly miss out on STBBI screening. Canada is presently facing an outbreak of syphilis in the Canadian Prairies and numerous challenges in timely screening of HCV-affected populations. This independent evaluation is relevant for such considerations.

Rapid test approvals can aid the achievement of elimination targets for syphilis, HCV, and HIV, particularly to meet the intensified demand for rapid testing in several provinces in a post–COVID-19 pandemic era. Demand for portable molecular platform-based tests is also high in high-volume clinics and community-based settings. While POC tests are useful to provide rapid results, molecular platforms combine the benefits of POC and conventional tests with DNA and RNA detection with a high diagnostic accuracy, and results could be provided for test-and-treat strategies without the need for further confirmation, offering a multitude of savings to the health care system [45,46]. This must be considered to reduce the burden of individuals living with undiagnosed infections in Canada and the world.

### Conclusions

We conclude that the AideSmart! integrated multiplexed STBBI rapid screening strategy offered by health aides was deemed acceptable, accurate, and feasible while impacting the detection of multiple STBBIs in a rapid turnaround time, all while satisfying end-user and health care provider preferences. This study demonstrated the utility of connected digital solutions and multiplexed POC testing assays with electronic readers, which can facilitate the detection of infections at the POC in challenging circumstances and among vulnerable populations. A total of 99.3% (398/401) of at-risk participants were tracked throughout the pandemic. The app-based strategy engaged participants and health care providers, demonstrating the relevance of digitally enabled diagnostic strategies in stressful pandemic times. While multiplexed assays with electronic readers provided accurate test results at the POC in a rapid turnaround time, impacting linkage to clinical decisions, the negative test results were also reassuring to health care providers involved in the care of these populations. This study is relevant not only for Canadians but also for global populations. Aligned with the WHO’s One Health approach [47], this digital strategy can be used to serve the hardest-to-reach undiagnosed marginalized populations worldwide to help meet elimination targets for triple infections.

## Abbreviations

DBS: dried blood spot
FIND: Foundation for Innovative New Diagnostics
HCV: hepatitis C virus
HIPAA: Health Insurance Portability and Accountability Act
POC: point-of-care
RECAP: Centre for Research, Education, and Clinical Care of At-Risk Populations
STBBI: sexually transmitted blood-borne infection
STD: sexually transmitted disease
STI: sexually transmitted infection
WHO: World Health Organization

## Data Availability

The datasets generated during and analyzed during this study are available from the corresponding author on reasonable request.
