# Correction: Feasibility, Preference, and Impact of a Rapid Multiplexed Point-of-Care Digital Innovation (AideSmart!) to Expedite Screening of Sexually Transmitted Blood-Borne Infections in At-Risk Populations in Canada: Cross-Sectional Study

**DOI:** 10.2196/67994

**Published:** 2024-10-28

**Authors:** Angela Karellis, Duncan Webster, Jean Boulanger, Kate Harland, Paige Feltmate, Stefanie Materniak, Gabriel Daunais-Laurin, Christine Mesa, Olivia Vaikla, John Kim, Nitika Pant Pai

**Affiliations:** 1 Infectious Diseases and Immunity in Global Health Research Institute of the McGill University Health Centre Montreal, QC Canada; 2 Centre for Research, Education & Clinical Care of At-Risk Populations Saint John, NB Canada; 3 REZO Montreal, QC Canada; 4 National Laboratory for HIV Reference Services Public Health Agency of Canada Winnipeg, MB Canada; 5 Faculty of Medicine McGill University Montreal, QC Canada

In “Feasibility, Preference, and Impact of a Rapid Multiplexed Point-of-Care Digital Innovation (AideSmart!) to Expedite Screening of Sexually Transmitted Blood-Borne Infections in At-Risk Populations in Canada: Cross-Sectional Study” (J Med Internet Res 2024;26:e55075), 2 errors were made during the publication process.

1) Affiliations 1 and 4:

1. Research Institute of the McGill University Health Centre, Montreal, QC, Canada

4. National Laboratory for HIV Reference Services, Winnipeg, MB, Canada

Have been revised to include subunits, as follows:

1. Infectious Diseases and Immunity in Global Health, Research Institute of the McGill University Health Centre, Montreal, QC, Canada

4. National Laboratory for HIV Reference Services, Public Health Agency of Canada, Winnipeg, MB, Canada

2) Tables 3 and 4 have been revised to adjust some numbers in “Sensitivity” and “Specificity” columns, as follows:

**Table 3:** Diagnostic accuracy of the Chembio and MedMira point-of-care (POC) tests in comparison to local laboratory tests in the AideSmart! cross-sectional multiplexed rapid sexually transmitted blood-borne infection screening study in Canada (2021-2022).

POC test		Sensitivity, % (95% CI)	Specificity, % (95% CI)
**HIV**			
	Chembio	100.00 (79.41-100.00)	98.71 (96.72-99.65)
	MedMira	100.00 (78.20-100.00)	100.00 (98.82-100.00)
**Syphilis**			
	Chembio	86.84 (71.91-95.59)	99.65 (98.06-99.99)
	MedMira	57.89 (40.82-73.69)	100.00 (98.72-100.00)
**Hepatitis C**			
	Chembio	—^a^	—
	MedMira	90.32 (80.12-96.37)	99.62 (97.92-99.99)

^a^Not applicable.

**Table 4:** Diagnostic accuracy of the Chembio and MedMira point-of-care (POC) tests in comparison to dried blood spot tests in the AideSmart! cross-sectional multiplexed rapid sexually transmitted blood-borne infection screening study in Canada (2021-2022).

POC test		Sensitivity, % (95% CI)	Specificity, % (95% CI)
**HIV**			
	Chembio	100.00 (76.84-100.00)	98.94 (97.30-99.71)
	MedMira	93.75 (69.77-99.84)	100.00 (99.02-100.00)
**Syphilis**			
	Chembio	80.00 (64.35-90.95)	99.03 (97.19-99.80)
	MedMira	55.00 (38.49-70.74)	100.00 (98.81-100.00)
**Hepatitis C**			
	Chembio	—^a^	—
	MedMira	91.15 (84.33-95.67)	99.29 (97.46-99.91)

^a^Not applicable.

The correction will appear in the online version of the paper on the JMIR Publications website on October 28, 2024 together with the publication of this correction notice. Because this was made after submission to PubMed and other full-text repositories, the corrected article has also been resubmitted to those repositories.

